# Disruption of the EmrAB-TolC efflux pump of *Escherichia coli* induces global metabolic changes in multiple growth conditions

**DOI:** 10.17912/micropub.biology.001437

**Published:** 2025-03-21

**Authors:** Dana E. Harmon, Klay Adamson, Joseph Wiersma

**Affiliations:** 1 Biology, California Lutheran University, Thousand Oaks, California, United States

## Abstract

Multidrug efflux pumps are transporters that are important for the removal of exogenous toxic molecules in bacteria. Recently, efflux pumps have been implicated in the regulation of metabolic homeostasis in *Escherichia coli* . Here, we investigated the contribution of EmrAB-TolC to metabolism in various conditions. Deletion of EmrB led to changes in several metabolic pathways, both in standard growth conditions and during nutrient stress. The pathways impacted include the tricarboxylic acid (TCA) cycle, and carbohydrate and amino acid metabolism. Our findings suggest that EmrAB-TolC contributes to maintaining metabolic homeostasis and adapts metabolism based on cellular needs.

**Figure 1. Deletion of emrB causes global metabolic changes in both rich and minimal media.**
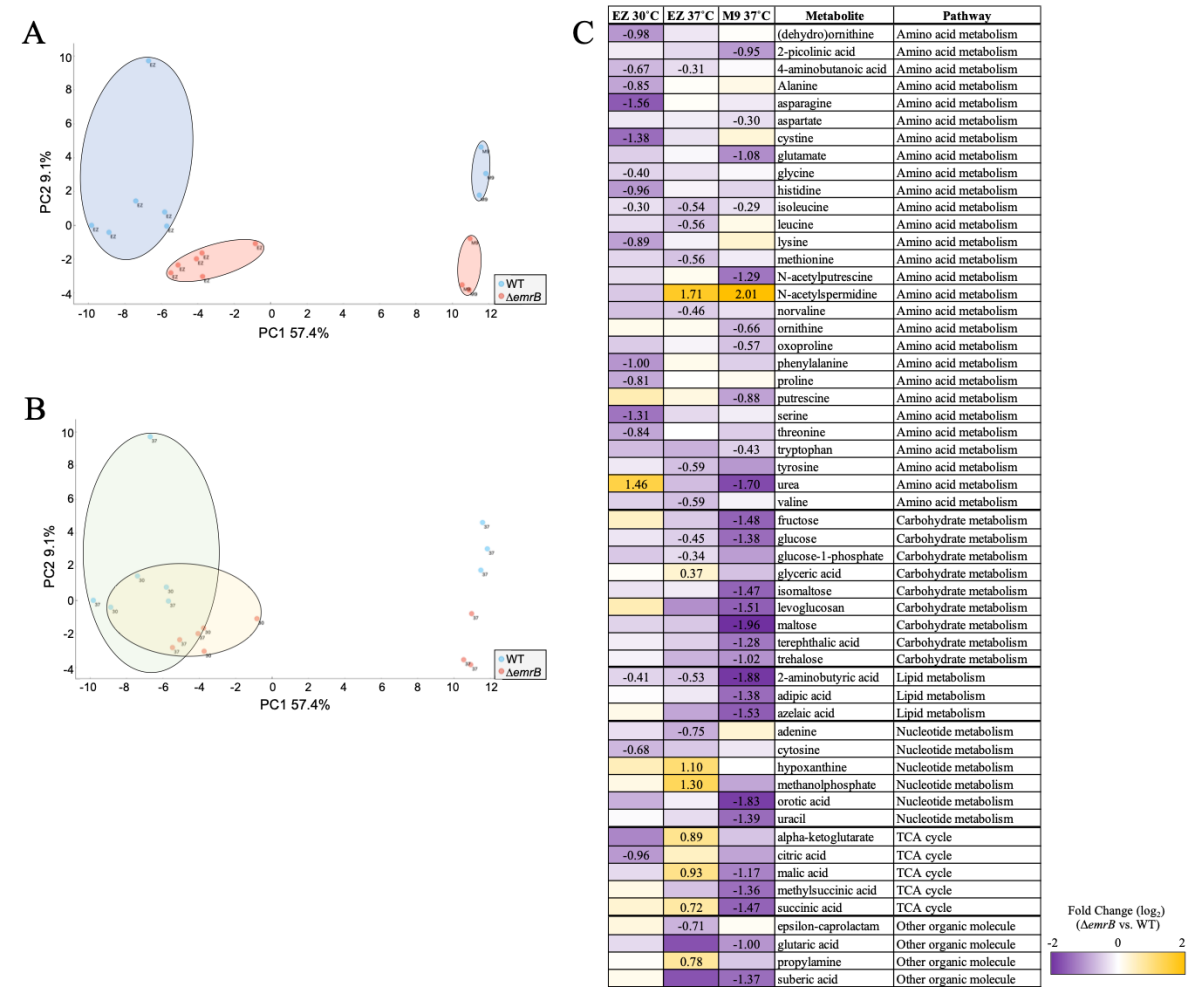
A. A Principle-component analysis (PCA) plot of the metabolomes of *E. coli* wild-type (WT) (blue) and ∆ *emrB* mutant (red) in EZ rich media (EZ) and minimal media (M9). The PCA plot displays the clustering of samples based on their metabolic profiles. B. The same PCA plot highlighting the effect of temperature (37˚C in green oval, 30˚C in yellow oval). C. Comparison of the ∆ *emrB* strain to the WT in different growth conditions shows changes in the accumulation of known cellular metabolites. This chart shows the fold change in the accumulation of metabolites in the ∆ *emrB* mutant versus the WT. Yellow color represents those metabolites that were overrepresented in the ∆ *emrB* strain; purple color represents those that are underrepresented. White color represents those metabolites whose accumulation was unchanged. The metabolites shown had statistically significant ( *p* -value ≤ 0.05) differences in at least one culture condition. The fold-difference between the ∆ *emrB* mutant and WT are shown (as log _2_ ) numerical values for those metabolites whose *p* -values were determined to be significant ( *p* ≤ 0.05). Metabolic pathway determinations were made using the publicly available Kyoto Encyclopedia of Genes and Genomes (KEGG; www.genome.jp/kegg) and EcoCyc (www.ecocyc.org) databases.

## Description

Efflux pumps are transporters found in all domains of life. Those found in bacteria are important for the extrusion of toxic molecules, such as bile salts, dyes, and various classes of antibiotics (Li et al., 2015). Because these pumps contribute to antibiotic resistance, they are often referred to as multidrug efflux pumps, referencing their well-characterized role in reducing the susceptibility of bacteria to antibiotics (Du et al., 2018; Li et al., 2015). The main and most well characterized multidrug efflux pump of *E. coli* , AcrAB-TolC, has been implicated in control of physiological processes such as metabolism, virulence, signaling, stress responses and motility (Cauilan et al., 2019; Harmon & Ruiz, 2022; Maldonado et al., 2023; Nishino et al., 2006; Padilla et al., 2010; Ruiz & Levy, 2014; Webber et al., 2009). Multidrug efflux pumps also exhibit extensive “functional redundancy” with different efflux pumps displaying overlapping substrate profiles, regulation, and possible roles in regulating homeostasis (Goetz et al., 2022; Teelucksingh et al., 2020; Teelucksingh et al., 2022) These observations suggest that other efflux pumps separate from AcrAB-TolC, may also play a role in the efflux of endogenous metabolites and contribute to maintenance of metabolic homeostasis in *E. coli* . However, relatively little is known about the endogenous functions of the other TolC-dependent efflux pumps. To address this gap in knowledge, we investigated the role of EmrAB-TolC in regulating the accumulation of metabolites.

The EmrAB-TolC efflux pump is a member of the major facilitator superfamily of efflux pumps found widely distributed in Gram-negative bacteria (Teelucksingh et al., 2020). This pump consists of three components: the substrate specificity subunit EmrB, which transports molecules across the inner membrane, the periplasmic adaptor protein EmrA, and TolC which acts as an outer membrane channel for EmrAB and a number of other efflux pumps found in *E. coli* (Teelucksingh et al., 2020; Yousefian et al., 2021). EmrAB-TolC has been implicated in resistance to several toxic molecules including dyes, antibiotics, bile salts, detergents, and proton motive force uncouplers (Furukawa et al., 1993; Lomovskaya & Lewis, 1992; Sulavik et al., 2001; Thanassi et al., 1997), as well as in the efflux of endogenously produced free fatty acids (Lennen et al., 2013).

Given that EmrAB-TolC has been previously demonstrated to be involved in the extrusion of free fatty acids (Lennen et al., 2013), we reasoned that this pump may also recognize and extrude other metabolites and/or contribute to global metabolism. Therefore, we used untargeted metabolomics to uncover metabolic changes that occur in a mutant of *E. coli* deleted for the substrate-binding subunit *emrB* (∆ *emrB* ) and compared it to the wild-type (WT) strain. We compared total metabolite levels in the extracts of cells grown in standard conditions (EZ rich media, 37˚C), as well as temperature-stress (EZ rich media, 30˚C), and nutrient-stress (M9 minimal media, 37˚C) growth conditions, to determine if the addition of temperature or nutrient stress revealed a specialized role for EmrAB-TolC in metabolism for these specific metabolic states.

The total levels of endogenous metabolites for both WT and ∆ *emrB* mutant *E. coli* were measured in the three media conditions, and the results compared using a primary component analysis ( Figure 1A ) to determine if the differences we observed in the metabolomics data were due to strain, media, or temperature differences. As anticipated, we found that media composition had a substantial impact on global metabolism, with cells grown in EZ rich media, having a distinct metabolome from cells grown in M9 minimal salts. We also observed that independent clustering of the WT and *emrB* -deleted strains suggests that the EmrAB-TolC efflux pump also exerts an influence on metabolism. Interestingly, we found a greater degree of overlap between the metabolic changes that occur between 30 and 37˚C in cells grown in rich media, suggesting a far smaller metabolic effect caused by temperature ( Figure 1B ).

Comparing the accumulation of metabolites between the WT and ∆ *emrB* mutant, we found that the ∆ *emrB* mutant had significant differences in the accumulation of several metabolites ( Figure 1C ). Notably, these metabolites varied in the different conditions, suggesting that the contribution of the pump changes in different metabolic circumstances. For example, in rich media at 37˚C, we found the statistically significant ( *p* ≤ 0.05) accumulation of three TCA cycle intermediates, including alpha-ketoglutarate (1.85-fold), succinic acid (1.65-fold), and malic acid (1.90-fold) in cell extracts. These findings suggest that the efflux activity of EmrAB-TolC, either directly or indirectly, controls the levels of metabolites in this critical central metabolic pathway in standard, non-stressed conditions. These findings are consistent with observations made for AcrAB-TolC, where it was shown that the TCA cycle intermediates fumaric acid and malic acid both accumulate in *E. coli* deleted for *acrB* (Cauilan et al., 2019).

In both rich and minimal media culture at 37˚C, we also observed accumulation of N8-spermidine in the ∆ *emrB* strain (3.3-fold and 4.0-fold, respectively). Previous reports have shown that the enzyme that produces this metabolite, spermidine- *N-* acetyltransferase, is more active in nutrient poor conditions (Fukuchi et al., 1994). Consistent with this, we observed a strong increase in total amounts of N8-spermidine produced by WT in EZ versus M9 (3.7-fold), as well as by the ∆ *emrB* mutant (4.6-fold) *.* As to why we observe an overaccumulation in the ∆ *emrB* strain versus the WT, remains unclear, but it has been shown that increased production of monoacetylated spermidine occurs when cells experience a variety of chemical and physical stresses (Carper et al., 1991). It is possible that the loss of EmrAB-TolC efflux activity leads a general stress response that induces the production of N8-spermidine in cells deleted for *emrB* or, more tantalizingly, acetylated spermidine may be a substrate of the EmrAB-TolC pump *.*

Here we present the findings of a metabolomics study, in which we investigated the contribution of the EmrAB-TolC efflux pump to metabolism in *E. coli* in standard, low-temperature, and nutrient-limited conditions. The authors of this study recognize that the complexity of biological systems requires the careful interpretation of metabolomic analysis findings. Therefore, any results from this study that suggest a role for EmrAB-TolC in the control of a specific metabolic pathway require further investigation to confirm these observations. Nevertheless, the global changes observed upon disruption of EmrAB-TolC provides compelling evidence for its ability to shape metabolite pools, and highlights the potential role of EmrAB-TolC as a regulator of metabolic homeostasis in both standard and nutrient limited conditions. This is the first metabolomics study investigating the EmrAB-TolC efflux pump and is the first in *E. coli* to examine how a single efflux pump regulates metabolism in multiple growth conditions. Our findings suggest that EmrAB-TolC is involved in shaping intracellular metabolite profiles, both in standard growth conditions and during nutrient limitation. This could be attributed to the ability of EmrAB-TolC to efflux substrates and/or intermediates from several metabolic pathways, including the TCA cycle, carbohydrate metabolism, and amino acid biosynthesis and degradation, with EmrAB-TolC effectively acting as a regulator of cellular metabolism. Alternatively, the metabolic changes we observed in this study may not be solely due to the direct loss of EmrAB-TolC efflux activity. Studies of AcrAB-TolC have shown that the periplasmic adapter protein AcrA can interact with two other efflux pumps, AcrD and AcrF, in addition to AcrB (Elkins & Nikaido, 2002, 2003). Therefore, in the absence of EmrB, it is possible that EmrA may have alternate associations with other pumps, which could lead to secondary effects on metabolism. While experimentally evaluating these possibilities goes beyond the scope of this study, these questions are important topics for follow up studies to further understand the complex interplay between the TolC-dependent efflux pumps and their impact on global metabolism. Other metabolomics studies, in addition to this one, suggest a broad role for efflux pumps as regulators of metabolism (Cauilan et al., 2019; Wang-Kan et al., 2021). The insights provided by this study of the EmrAB-TolC efflux pump provides further evidence for the significant impact the TolC-dependent efflux pumps play in the ability of *E. coli* to adapt to diverse environments.

## Methods

*Strains*

The DH8003 was generated from JW2661 (Keio collection; (Baba et al., 2006)) by removal of the *kan* cassette as described previously (Datsenko & Wanner, 2000).

*Untargeted metabolomics*

Three biological replicates of the parental and ∆ *emrB* mutants were cultured with aeration in either EZ rich defined medium supplemented with 0.2% glucose (Teknova) at either 37˚C or 30˚C, or in M9 minimal salts (MP Biomedicals) supplemented with 0.2% glucose and 1 mM MgSO _4_ , at 37˚C to mid-exponential (OD 600 of 0.35-0.4). Two milliliters of each culture were flash frozen in liquid nitrogen and shipped on dry ice to the West Coast Metabolomics Center (WCMC) at UC Davis for analysis by untargeted metabolomics. The cell extracts were prepared using the Matyash extraction procedure (Matyash et al., 2008), derivatized with MSTFA/FAMEs, and analyzed using the WCMC ALEX-CIS GCTOF MS (automated liner exchange-cold injection system-gas chromatography time of flight-mass spectrometry) platform. Quantification of each metabolite was reported by WCMC as peak height, following the established protocol within the BinBase (rtx5) algorithm established by the core facility. We further normalized the peak heights for all metabolites to the optical density (600 nm) measurements for the individual samples from which the peaks were derived. Finally, we determined the average peak height for each metabolite and present the data as the log _2_ -fold difference between the ∆ *emrB* mutant and the WT. To determine statistical significance, we used the student’s t-test (two independent samples with equal variance and a two tailed distribution) in Microsoft Excel 365 to compare the abundance of a given metabolite in the ∆ *emrB* to that found in the WT. Those that had a *p-* value ≤ 0.05 were considered significant.

## Reagents

**Table:** 

**Strains**	**Genotype**	**Source/Reference**
BW25113	(parental) F-λ-Δ *(araD–araB)567 ΔlacZ4787(::rrnB-3) rph-1* Δ *(rhaD-rhaB)568 hsdR514*	CGSC, Keio collection (Baba 2006)
DH8003	BW25113 ∆ *emrB*	This study

## Data Availability

Description: Full metabolomics data and analysis. Resource Type: Dataset. DOI: https://doi.org/10.22002/f019j-q9e75

## References

[R1] Baba T, Ara T, Hasegawa M, Takai Y, Okumura Y, Baba M, Datsenko KA, Tomita M, Wanner BL, Mori H (2006). Construction of Escherichia coli K-12 in-frame, single-gene knockout mutants: the Keio collection.. Mol Syst Biol.

[R2] Carper SW, Willis DG, Manning KA, Gerner EW (1991). Spermidine acetylation in response to a variety of stresses in Escherichia coli.. J Biol Chem.

[R3] Cauilan A, Ramos K, Harmon DE, Ruiz C (2019). Global effect of the AcrAB-TolC multidrug efflux pump of Escherichia coli in cell metabolism revealed by untargeted metabolomics.. Int J Antimicrob Agents.

[R4] Datsenko KA, Wanner BL (2000). One-step inactivation of chromosomal genes in Escherichia coli K-12 using PCR products.. Proc Natl Acad Sci U S A.

[R5] Du D, Wang-Kan X, Neuberger A, van Veen HW, Pos KM, Piddock LJV, Luisi BF (2018). Multidrug efflux pumps: structure, function and regulation.. Nat Rev Microbiol.

[R6] Elkins CA, Nikaido H (2002). Substrate specificity of the RND-type multidrug efflux pumps AcrB and AcrD of Escherichia coli is determined predominantly by two large periplasmic loops.. J Bacteriol.

[R7] Elkins CA, Nikaido H (2003). Chimeric analysis of AcrA function reveals the importance of its C-terminal domain in its interaction with the AcrB multidrug efflux pump.. J Bacteriol.

[R8] Fukuchi J, Kashiwagi K, Takio K, Igarashi K (1994). Properties and structure of spermidine acetyltransferase in Escherichia coli.. J Biol Chem.

[R9] Furukawa H, Tsay JT, Jackowski S, Takamura Y, Rock CO (1993). Thiolactomycin resistance in Escherichia coli is associated with the multidrug resistance efflux pump encoded by emrAB.. J Bacteriol.

[R10] Goetz JA, Kuehfuss NM, Botschner AJ, Zhu S, Thompson LK, Cox G (2022). Exploring functional interplay amongst Escherichia coli efflux pumps.. Microbiology (Reading).

[R11] Harmon DE, Ruiz C (2022). The Multidrug Efflux Regulator AcrR of Escherichia coli Responds to Exogenous and Endogenous Ligands To Regulate Efflux and Detoxification.. mSphere.

[R12] Lennen RM, Politz MG, Kruziki MA, Pfleger BF (2012). Identification of transport proteins involved in free fatty acid efflux in Escherichia coli.. J Bacteriol.

[R13] Li XZ, Plésiat P, Nikaido H (2015). The challenge of efflux-mediated antibiotic resistance in Gram-negative bacteria.. Clin Microbiol Rev.

[R14] Lomovskaya O, Lewis K (1992). Emr, an Escherichia coli locus for multidrug resistance.. Proc Natl Acad Sci U S A.

[R15] Maldonado J, Czarnecka B, Harmon DE, Ruiz C (2023). The multidrug efflux pump regulator AcrR directly represses motility in Escherichia coli.. mSphere.

[R16] Nishino K, Latifi T, Groisman EA (2006). Virulence and drug resistance roles of multidrug efflux systems of Salmonella enterica serovar Typhimurium.. Mol Microbiol.

[R17] Padilla E, Llobet E, Doménech-Sánchez A, Martínez-Martínez L, Bengoechea JA, Albertí S (2009). Klebsiella pneumoniae AcrAB efflux pump contributes to antimicrobial resistance and virulence.. Antimicrob Agents Chemother.

[R18] Ruiz C, Levy SB (2013). Regulation of acrAB expression by cellular metabolites in Escherichia coli.. J Antimicrob Chemother.

[R19] Sulavik MC, Houseweart C, Cramer C, Jiwani N, Murgolo N, Greene J, DiDomenico B, Shaw KJ, Miller GH, Hare R, Shimer G (2001). Antibiotic susceptibility profiles of Escherichia coli strains lacking multidrug efflux pump genes.. Antimicrob Agents Chemother.

[R20] Teelucksingh T, Thompson LK, Cox G (2020). The Evolutionary Conservation of Escherichia coli Drug Efflux Pumps Supports Physiological Functions.. J Bacteriol.

[R21] Teelucksingh T, Thompson LK, Zhu S, Kuehfuss NM, Goetz JA, Gilbert SE, MacNair CR, Geddes-McAlister J, Brown ED, Cox G (2022). A genetic platform to investigate the functions of bacterial drug efflux pumps.. Nat Chem Biol.

[R22] Thanassi DG, Cheng LW, Nikaido H (1997). Active efflux of bile salts by Escherichia coli.. J Bacteriol.

[R23] Wang-Kan X, Rodríguez-Blanco G, Southam AD, Winder CL, Dunn WB, Ivens A, Piddock LJV (2021). Metabolomics Reveal Potential Natural Substrates of AcrB in Escherichia coli and Salmonella enterica Serovar Typhimurium.. mBio.

[R24] Webber MA, Bailey AM, Blair JM, Morgan E, Stevens MP, Hinton JC, Ivens A, Wain J, Piddock LJ (2009). The global consequence of disruption of the AcrAB-TolC efflux pump in Salmonella enterica includes reduced expression of SPI-1 and other attributes required to infect the host.. J Bacteriol.

[R25] Yousefian N, Ornik-Cha A, Poussard S, Decossas M, Berbon M, Daury L, Taveau JC, Dupuy JW, Đorđević-Marquardt S, Lambert O, Pos KM (2020). Structural characterization of the EmrAB-TolC efflux complex from E. coli.. Biochim Biophys Acta Biomembr.

